# Novel Brain-Penetrant, Small-Molecule Tubulin Destabilizers for the Treatment of Glioblastoma

**DOI:** 10.3390/biomedicines12020406

**Published:** 2024-02-09

**Authors:** Lilian A. Patrón, Helen Yeoman, Sydney Wilson, Nanyun Tang, Michael E. Berens, Vijay Gokhale, Teri C. Suzuki

**Affiliations:** 1 Reglagene, Inc., Tucson, AZ 85719, USA; patron@reglagene.com (L.A.P.); yeoman@reglagene.com (H.Y.); gokhale@reglagene.com (V.G.); 2 Translational Genomics Research Institute (TGen), Phoenix, AZ 85004, USA; ntang@tgen.org (N.T.); mberens@tgen.org (M.E.B.)

**Keywords:** glioblastoma, brain cancers, small molecules, microtubules, tubulin destabilizer

## Abstract

Glioblastoma (GB) is the most lethal brain cancer in adults, with a 5-year survival rate of 5%. The standard of care for GB includes maximally safe surgical resection, radiation, and temozolomide (TMZ) therapy, but tumor recurrence is inevitable in most GB patients. Here, we describe the development of a blood–brain barrier (BBB)-penetrant tubulin destabilizer, RGN3067, for the treatment of GB. RGN3067 shows good oral bioavailability and achieves high concentrations in rodent brains after oral dosing (C_max_ of 7807 ng/mL (20 μM), T_max_ at 2 h). RGN3067 binds the colchicine binding site of tubulin and inhibits tubulin polymerization. The compound also suppresses the proliferation of the GB cell lines U87 and LN-18, with IC_50_s of 117 and 560 nM, respectively. In four patient-derived GB cell lines, the IC_50_ values for RGN3067 range from 148 to 616 nM. Finally, in a patient-derived xenograft (PDX) mouse model, RGN3067 reduces the rate of tumor growth compared to the control. Collectively, we show that RGN3067 is a BBB-penetrant small molecule that shows in vitro and in vivo efficacy and that its design addresses many of the physicochemical properties that prevent the use of microtubule destabilizers as treatments for GB and other brain cancers.

## 1. Introduction

Glioblastoma (GB) or grade IV glioma (WHO classification) is an aggressive brain tumor of astrocytic lineage that is inevitably recurrent. It is one of the most common malignant primary brain tumors, with an incidence of 5 per 100,000 [1]. The median survival time post-diagnosis is approximately 15 months, with a 5-year survival rate of only 5%. The current standard of care is maximally safe surgical resection, followed by radiation and TMZ chemotherapy; however, tumor recurrence occurs in almost all patients, usually 6–9 months after treatment [2]. The disease outcome has not improved for several decades, and an urgent need for novel therapies exists.

Microtubule targeting agents (MTAs) have long been an important class of anticancer drugs. Microtubules (MTs), the backbone of the cytoskeleton, are involved in multiple cellular processes, including cell migration and mitosis. MTs are polymers consisting of α- and β-tubulin heterodimers and associated proteins. MTAs disrupt polymer dynamics, triggering cell cycle arrest that leads to cell death. Five distinct sites on the β-tubulin subunit have been targeted by MTAs, and an additional binding site has been identified on α-tubulin. MTAs can be categorized into those that inhibit polymerization (tubulin destabilizers, e.g., vinca alkaloids and colchicine) and those that inhibit depolymerization (tubulin stabilizers, e.g., taxanes and epothilones) [3]. In the clinic, they are utilized as both a first- and second-line standard of care in a variety of solid and hematological malignancies that lack targeted therapies. They are typically administered in combination with other drugs to achieve a greater therapeutic window and are approved for use with radiation, DNA-damaging agents, and immune checkpoint inhibitors.

Despite the success of MTAs as anticancer drugs, their clinical use is limited by poor pharmacokinetics, toxicity, and the development of drug resistance. The high molecular weight and lack of oral bioavailability of MTAs require intravenous administration, resulting in high peak drug concentrations that likely contribute to their associated neuropathic and myeloid toxicities. Their failure to cross the BBB has excluded them from the treatment arsenal for high-grade gliomas (HGGs) and other central nervous system (CNS) cancers, including brain metastases. HGGs have been shown to be sensitive to MTAs in vitro [4]. Furthermore, glioma tumorigenesis is mediated by neurite-like protrusions, a MT-mediated intercellular process [5]. Thus, the development of BBB-penetrant MTAs could be a paradigm shift in the treatment of this disease.

The development of resistance to MTAs is mediated by several mechanisms. First, the aberrant expression of β-tubulin isotypes, particularly Class III β-tubulin, is associated with resistance and tumor aggressiveness, forming part of a complex pro-survival pathway that is poorly understood [6]. A second important resistance mechanism is the overexpression of membrane-bound P-glycoprotein (P-gp) pumps, resulting in drug efflux [7]. MTAs that bind to the colchicine binding site on β-tubulin are less susceptible to P-gp pump efflux, but to date, they have not been approved as anticancer therapeutics due to toxicities associated with their specific chemical class [8].

Herein, we describe the identification of a series of small molecules that address many of the shortcomings of known MTAs. To improve the drug-like and physiochemical properties of our molecules, we designed and synthesized a series of analogs optimized for low molecular weight, low polar surface area, and a good CNS multiparametric optimization (MPO) score [9]. The MPO score uses six calculated properties of the molecule—molecular weight, clog D, clog P, number of hydrogen bond donors (HBDs), pKa and topological polar surface area. These properties for each molecule are transformed into a score from 0 to 1. Scores are added to arrive at an MPO score ranging from 0 to 6 for each molecule. One of these novel molecules, RGN3067, has excellent in vitro early absorption, distribution, metabolism, and elimination (eADME) properties (including solubility, microsomal stability, and cellular permeability). RGN3067 has activity against human HGG cell lines (including the TMZ-resistant cell line LN-18), achieving nanomolar inhibition in a cell viability assay. Additionally, in mice, oral administration of RGN3067 inhibits tumor growth in an HGG PDX model. RGN3067 shows minimal in vivo toxicity and achieves equal levels in plasma and brain.

The results presented here provide the basis for ongoing and future in vivo studies to further evaluate the potential of orally available, BBB-penetrant MTAs for both HGGs and other CNS cancers that currently have limited therapeutic options.

## 2. Materials and Methods

### 2.1. Cell Culture

U87 glioblastoma (ATCC HTB-14; Manassas, VA, USA) cells were grown in DMEM/10% FCS, LN-18 glioblastoma cells (ATCC CRL-2610; Manassas, VA, USA) were grown in DMEM/5% FCS, HMC3 microglial cells (ATCC CRL-3304; Manassas, VA, USA) were grown in EMEM/10% FCS, and MCF-7 mammary carcinoma cells (ATCC HTB-22; Manassas, VA, USA) were grown in RPMI/10% FCS. Patient-derived, low-passage, genetically heterogeneous primary cells GBM12, GBM15, GBM39 and GBM43 (established in Mayo Clinic Hospital) were cultured in serum-free DMEM/F12 (Cytiva HyClone; Logan, UT, USA) with 20 ng/mL EGF, 20 ng/mL FGF, 2% B12, 1% N2, and 1% gentamycin solution. Cells were cultured at 37 °C in 5% CO_2_.

### 2.2. Cell Viability Assay

Cells (100 µL/well) were seeded in 96-well tissue culture plates (Greiner Bio-One 650090; Kremsmünster, Austria). The plating density (cells/well) for each cell line was as follows: LN-18 (3000), U87 (4000), and HMC3 (5000). After overnight incubation, compounds were added to the cells (50 µL at 3× concentration, final 0.1% DMSO), and plates were incubated for 72 h. Cell viability was assessed by the addition of 25 µL of alamarBlue for a final concentration of 100 µM final concentration (Resazurin redox reagent, Sigma-Aldrich R7017; St. Louis, MO, USA). After a 4 h incubation, fluorescence was read on a CLARIOstar Plus plate reader (BMG Labtech, Ortenberg, Germany) at EX 570/EM 590 nm.

Short-term cultures of patient-derived cells GBM12, GBM15, GBM39 and GBM43 (used with permission Mayo Foundation) were seeded (1000 cells/well) in solid white Corning 384-well plates (Corning, NY, USA) and cultured overnight. Cells were treated with DMSO or RGN3067 in a 13-point dose response with a 1:3 serial dilution from an initial concentration of 100 μM. After 144 h, cell viability was measured using CellTiter-Glo (Promega, Madison, WI, USA). Data were normalized to DMSO control, and absolute IC_50_ values were calculated using GraphPad Prism 10 (San Diego, CA, USA).

### 2.3. Cell Cycle Analysis

Cells (400,000) were seeded into T25 tissue culture flasks and treated with compound for 24 h prior to harvesting with TrypLE (Thermo Fisher Scientific 12605036; Waltham, MA, USA). Cells were washed in ice-cold PBS, resuspended in 1 mL of ice-cold 70% ethanol, and fixed overnight at −20 °C. Cells were centrifuged and resuspended in PBS containing RNAse A at a final concentration of 0.5 mg/mL (MilliporeSigma 10109142001; Burlington, MA, USA) and propidium iodide at a final concentration of 0.04 mg/mL (Sigma-Aldrich P4170, St. Louis, MO, USA) for 1 h at 37 °C. Nuclear staining was analyzed using a BD FACSCanto II flow cytometer (BD Biosciences; Franklin Lakes, NJ, USA) to determine cell cycle distribution. Cell cycle analysis was performed using the BD FACSDiva software v8.0.

### 2.4. Immunofluorescent Staining of Cellular β-Tubulin

U87 cells (4000/well) were plated in black 96-well plates (Perkin Elmer 6055302; Waltham, MA, USA) and grown overnight. Cells were treated with compounds for 24 h, fixed with 4% paraformaldehyde for 20 min, and permeabilized with FoxP3 permeabilization buffer (eBioscience/Thermo Fisher Scientific 00-5523-00; Waltham, MA, USA) for 10 min at RT. Cells were incubated with an anti-TUBB3 (Tuj1) antibody (1:1000; STEMCELL Technologies 60052; Vancouver, BC, Canada) labeled with Hoechst 33342 dye (1:2000; Thermo Fisher Scientific H3570; Waltham, MA, USA). Images (40×) were taken with an automated high-content imaging microscope (Operetta, Perkin Elmer; Waltham, MA, USA).

### 2.5. Tubulin Polymerization Assay

Tubulin polymerization was evaluated by monitoring the incorporation of a fluorescent reporter into MTs during polymerization. The tubulin polymerization assay kit (Cytoskeleton, Inc. BK011P; Denver, CO, USA) was used according to the manufacturer’s instructions. Briefly, the tubulin reaction mix was kept on ice until immediately prior to use. The compound (5 µL/well) was added to a half-area 96-well plate (Corning Costar 3686; Corning, NY, USA) and incubated for 1 min in the CLARIOstar Plus plate reader (BMG Labtech; Ortenberg, Germany) at 37 °C before the addition of 50 µL of the tubulin reaction mix/well. Fluorescence was read every min for 160 min at EX 350/EM 435 nm.

### 2.6. Colchicine Competitive Binding Assay

Porcine tubulin (20 µM, Cytoskeleton, Inc., T240; Denver, CO, USA) was incubated with compounds for 45 min at 37 °C in 80 mM PIPES pH 6.9, 2 mM MgCl_2_, 0.5 mM EGTA, 0.2 mM GTP prior to the addition of colchicine for a 10 µM final concentration (Selleckchem S2284; Houston, TX, USA). After a 45 min incubation, the fluorescence of the colchicine–tubulin complex was measured at EX 380/EM 435 nm with the CLARIOstar Plus plate reader (BMG Labtech; Ortenberg, Germany).

### 2.7. Colchicine-Binding Site Cellular Assay

*N,N′*-ethylene-bis (iodoacetamide) (EBI) cross-links cysteines 239 and 354 in the colchicine binding pocket of β-tubulin, the formation of which can be inhibited by colchicine binding site molecules. A total of 1.5 × 10^6^ MCF-7 cells were seeded overnight in a T25 flask and pre-incubated with compounds for 2 h, then treated with EBI (Abcam ab144980; Cambridge, MA, USA) for 1.5 h at 37 °C. Cells were washed with PBS and lysed for 30 min at 4 °C in RIPA buffer supplemented with a protease inhibitor cocktail (Sigma-Aldrich P8340; St. Louis, MO, USA). Lysates were collected by centrifugation (12,000× *g*, 4 °C, 30 min) and stored at −80 °C until detection of the EBI: β-tubulin adduct by Western blot. Briefly, 20 µg of each sample was loaded on a 10% Mini-Protean TGX SDS gel (Bio-Rad 4561034; Hercules, CA, USA). Proteins were transferred to nitrocellulose membranes (iBlot dry blotting system, Invitrogen; Waltham, MA, USA) and blocked for 1 h post-transfer (Intercept blocking buffer, Li-COR Biosciences 927-80001; Lincoln, NE, USA). Primary antibodies were incubated overnight at 4 °C (mouse anti-β-tubulin, Sigma-Aldrich T4026, St. Louis, MO, USA; rabbit anti-GAPDH, Cell Signaling Technology 14C10, Danvers, MA, USA) in blocking buffer/0.2% Tween 20. Membranes were washed twice (PBS/0.1% Tween 20) and incubated for 1 h at RT with secondary antibodies (IRDye 680 anti-mouse, Li-COR Biosciences 926-68072; IRDye 800 anti-rabbit, Li-COR Bioscience 926-32211; Lincoln, NE, USA) in blocking buffer/0.1% Tween 20. After three washes, membranes were dried for 2 h prior to imaging on the Odyssey CLx Imager (Li-COR Biosciences; Lincoln, NE, USA). Band intensities were quantified by densitometric analysis using Fiji/ImageJ software (NIH) v2.3.0.

### 2.8. Reversibility Assay

U87 cells were treated with increasing concentrations (up to 10 μM) of RGN3067 and the control compounds colchicine (Selleckchem S2284; Houston, TX, USA) and sabizabulin (MedChem Express HY-120599; Monmouth Junction, NJ, USA) and cultured for 6 h. Compounds were either washed out or left in the medium. Cells were then cultured for an additional 72 h and cell viability was examined using CellTiter-Glo (Promega, Madison, WI, USA).

### 2.9. Compound Kinetic Solubility

Kinetic solubility was determined in phosphate buffer. The compound (4 µL, 10 mM DMSO) was added to the buffer (396 μL 100 mM phosphate buffer, final 1% DMSO) and shaken at 1000 rpm at RT for 1 h. Samples were filtered (Millipore MSSLBPC10; Burlington, MA, USA), the primary filtrate was discarded, and the subsequent filtrate was collected. The supernatant solution was diluted 10× with DMSO. Test samples (10 µL) and working standard curve samples were added to the stop solution (100 μL, containing internal standard) and water/0.1% FA (100 μL). Samples were analyzed by LC-MS/MS. Albendazole, propranolol, and 4,5-diphenylimidazole were used as positive controls.

### 2.10. Compound Microsomal Stability

Compounds (5 µL, 20 mM DMSO) were added to 195 µL of methanol to obtain a 500 µM spiking solution. Spiking solution (1.5 μL) and liver microsomes (18.75 μL, 20 mg/mL) were added to Buffer C (479.75 μL) on ice. Plates were pre-incubated at 37 °C for 5 min. At 0 min, spiking/microsome solution (30 μL) was added to the precipitation solution (400 μL) and NADPH stock solution (6 mM in Buffer C, 0.1 M PBS, pH 7.4) on ice. At additional time points, spiking/microsome solution (180 μL) was added to the assay plates, and the reaction initiated with the NADPH stock solution (90 μL, 6 mM). Plates were sealed and incubated at 37 °C. At 5, 15, 30 and 60 min time points, aliquots of the microsomal incubation system (45 μL) were removed and added to precipitation solution (400 μL). Supernatants were evaluated by LC-MS/MS. Ketanserin was used as a positive control. All reactions were performed in duplicate.

### 2.11. Plasma Protein Binding Studies

Compounds (5 µM/0.2% DMSO) were tested in dialysis chambers. Blank dialysis buffer was added to the receiver side (100 μL), and plasma (100 μL) with test and reference compounds was added to the donor side of the chamber and incubated at 37 °C on an orbital shaker. Plasma was precipitated at 0 and 5 h. Aliquots from donor and receiver sides of the dialysis apparatus (25 μL) were added to sample preparation plates and mixed with aliquots of the opposite matrix (25 μL, blank buffer to plasma and vice versa). Samples were quenched with stop solution (400 μL) containing internal standard, vortexed at 600 rpm for 10 min, and centrifuged at 4000 rpm for 10 min. Supernatants were analyzed by LC-MS. Quinidine and warfarin were used as positive controls.

### 2.12. Cellular Permeability of RGN3067

Cellular permeability was tested using the MDR1-MDCK (multidrug-resistant-1, Madin–Darby canine kidney) cell line. Donor solution was added to a 96-well plate (2 μL, 2 mM compound; 6 μL DMSO in 1992 μL of Hanks’ balanced salt solution [HBSS] buffer) and shaken at 1000 rpm for 10 min (final 0.4% DMSO). Transepithelial electrical resistance (TEER) values were used as a quality control check for monolayer integrity. At 3–5 days post-seeding, each MDCK II cell monolayer was tested to ensure the TEER value was ≥ 83 Ω⋅cm^2^. The compound donor working solution was centrifuged (4000 rpm for 5 min) to remove any precipitated compound prior to loading donor chambers. Apical and basolateral plates were warmed at 37 °C for about 5 min prior to the initiation of transport (37 °C, 90 min).

Luciferin marker Lucifer Yellow (150 μL, 50 μM Lucifer Yellow in HBSS, pH 7.4) was added to the apical compartment, and transfer buffer (600 μL) was added to the receiver plate (basolateral compartment) and incubated at 37 °C for 60 min. Apical Lucifer Yellow concentrations for LYT0 and LYT60 were measured by a fluorometer (EX 485/EM 535 nm). Donor and receiver samples were analyzed by LC-MS. Metoprolol, enalapril, and quinidine were used as positive controls. The efflux ratio was calculated as the ratio of apparent permeability (basal-to-apical) and apparent permeability (apical-to-basal).

### 2.13. Pharmacokinetics of RGN3067

Pharmacokinetic studies were carried out by DMPK at WuXi AppTec, Inc. (Cranbury, NJ, USA). The pharmacokinetics of RGN3067 were evaluated in three male CD-1 mice. RGN3067 formulated as a 10 mg/mL solution (20% DMSO, 80% PEG400: PVP 95:5) was administered by oral gavage at a dose of 100 mg/kg. Plasma samples were drawn at 0.5, 1, 2, 4, 6, 10 and 24 h. Samples were analyzed by LC-MS/MS. A calibration curve was established using Waters ACQUITY UPLC BEH C18 column and formic acid in water and formic acid in acetonitrile as mobile phase. Tolbutamide and labetalol were used as internal standards for LC-MS/MS analysis. Compound was detected using electrospray ionization (ESI) positive detection. Pharmacokinetic parameters were calculated using the PO-noncompartmental linear model in Phoenix WinNonlin software v8.3 (Certara, Princeton, NJ, USA).

### 2.14. Brain Pharmacokinetics of RGN3067

Brain pharmacokinetic studies were carried out by DMPK at WuXi AppTec, Inc. (Cranbury, NJ, USA). The brain penetrance of RGN3067 was evaluated in three male CD-1 mice. RGN3067 was formulated as a 10 mg/mL solution (20% DMSO, 80% PEG400: PVP 95:5) and administered by oral gavage at a dose of 100 mg/kg. Plasma and brain samples were collected at 1, 4, and 8 h. Samples were analyzed by LC-MS/MS. A calibration curve was established using Waters ACQUITY UPLC BEH C18 column and formic acid in water and formic acid in acetonitrile as mobile phase. Tolbutamide and labetalol were used as internal standards for LC-MS/MS analysis. Compound was detected using electrospray ionization (ESI) positive detection. Pharmacokinetic parameters were calculated using the PO-noncompartmental linear model in Phoenix WinNonlin software v8.3 (Certara, Princeton, NJ, USA).

### 2.15. Maximum Tolerated Dose Study with RGN3067

RGN3067 was administered to athymic female mice (4–6 weeks old) twice daily for 5 days by oral gavage at doses of 25, 50, 100 or 200 mg/kg (BID; 6 mice per group, including vehicle control). One mouse from each group was euthanized for PK analysis at 6 h post-initial dose. The weight and physical states of the animals were monitored during the 5 days of treatment and for 5 days following drug administration. The maximum tolerated dose was defined as the dose at which none of the mice within the cohort lost more than 20% body weight during the 10-day study and was used for subsequent efficacy testing.

### 2.16. The Efficacy of RGN3067 in a PDX Model of GB

Animal protocols were approved by the Animal Care and Use Committee (ACUC) of the institutes and CRO labs. All studies were performed in compliance with the guidelines. GBM12 cells (5 × 10^6^) were injected subcutaneously into athymic nude female mice aged 4–6 weeks. Subcutaneous (SC) tumor size was measured using an external caliper. The greatest longitudinal diameter (length) and the greatest transverse diameter (width) were determined while mice were scruffed and conscious. Tumor volume was calculated by the modified ellipsoidal formula: V = ½ (Length × Width^2^) [10]. RGN3067 solution was prepared for each mouse. RGN3067 (9.91 mg) was dissolved in DMSO (17 µL) by vortexing for 30–60 s. Approximately 69 μL of PEG400: PVP (95:5, Sigma-Aldrich; St. Louis, MO, USA) solution was added and vortexed.

After tumors reached 100–150 mm^3^, mice were dosed with RGN3067 solution (43 µL, 100 mg/kg) or vehicle control twice daily by oral gavage (total dose was 200 mg/kg). Mice were dosed using cycles of 5 days on/2 days off, for a total of four cycles. Mice were euthanized once the tumor reached 2000 mm^3^.

### 2.17. Metabolite Profiling of RGN3067 in Hepatocytes

Metabolite profiling was carried out by WuXi DMPK (Nanjing, China). RGN3067 (10 μM) was profiled in CD-1 mice (BioIVT, Westbury, NY, USA), SD rats (BioIVT), beagle dogs (BioIVT), cynomolgus monkeys (Research Institute for Liver Diseases [RILD], Shanghai, China), and human cryopreserved hepatocytes (BioIVT). The positive control was 7-ethoxycoumarin (30 μM). Compounds were added to hepatocytes (400 µL, 1 × 10^6^/mL), and reactions were carried out at 37 °C in 5% CO_2_/saturated humidity for 120 min. Reactions were stopped by the addition of ice-cold ACN (400 μL). Samples were shaken at 300 rpm for 10 min, then centrifuged at 13,000× *g* for 10 min. Supernatants were evaporated under a stream of N_2_. Residue from the RGN3067 incubation was reconstituted with ACN: H_2_O (200 μL, *v*:*v*, 3:7), followed by centrifugation at 14,000 rpm for 10 min, and the supernatant was used for qualitative and semi-quantitative analysis using LC-UV/HRMS. A Shimadzu UFLC-20A with autosampler was used for chromatographic separation. Detection and structure were determined using a Thermo Q-Extractive mass spectrometer in full MS/MS^2^ scan mode and Xcalibur Data Acquisition and Interpretation Software v4.2.

### 2.18. Data Processing

All statistical analyses were performed using GraphPad Prism v10 (San Diego, CA, USA). Absolute IC_50_ values were determined by a nonlinear regression model. Tests to determine significance are detailed in the relevant figure and table legends. Briefly, the Shapiro–Wilk test was used for testing the normality of the data. Unpaired t-tests or one-way ANOVAs were used to determine the statistical significance of treatment conditions. Asterisks denote significance (* *p* < 0.05; ** *p* < 0.01; *** *p* < 0.001, **** *p* < 0.0001).

## 3. Results

### 3.1. The Chemistry of RGN3067 and Related Compounds

Table 1 shows the structures and properties of the compounds used in the study. All compounds (RGN3067, RGN3062, and RGN3096) were synthesized using a three-step synthesis starting from cycloalkyl amides. The final compounds were analyzed using proton nuclear magnetic resonance (^1^H-NMR), high-performance liquid chromatography (HPLC), and mass spectral characterization before testing (see Supplementary Materials).

These compounds have two sites for structural variations. Cyclohexyl and cyclobutyl, substituted with a methoxy group, were selected at the carbonyl urea position. Chloropyridine and chloropyrimidine groups were used in the aryloxy position. All three compounds have molecular weights below 425 and a calculated LogD of less than 3, resulting in favorable CNS MPO scores greater than 4.0. RGN3062 and RGN3096 are cis and trans isomers, as indicated by their ^1^H-NMR spectra. These isomers differ in the orientation of two protons on the cyclohexyl ring. RGN3067 was selected as our lead molecule based on its physicochemical properties and in vitro antitumor cell activity. The isomer pair was included to explore the stereochemical selectivity of our molecules.

### 3.2. The Solubility, Plasma Protein Binding, and Blood–Brain Permeability of RGN3067

The eADME properties of RGN3067 are shown in Supplementary Table S1. RGN3067 exhibits moderate kinetic solubility (5.7 µM) and strong plasma protein binding with values ranging from 97.4 to 98.2% for mouse, rat, and human species. BBB penetration is the major obstacle for effective small-molecule therapeutics for glioma, as P-gp efflux pumps in the BBB can prevent the absorption of small molecules into the brain. We utilized Madin–Darby canine kidney cells that overexpress multidrug resistance pumps to screen compounds for BBB permeability. We found that RGN3067 showed a good apparent permeability coefficient of 22.7 × 10^−6^ cm/s and an efflux ratio of 0.61 (Supplementary Table S1), indicating that it is not a substrate for MDR1 efflux pumps and that it likely has high BBB permeability.

### 3.3. In Vitro Metabolism and Metabolite Identification of RGN3067

As a first step in understanding the metabolic stability of RGN3067, its half-life was determined in mouse, rat, and human liver microsomes. The half-life in mice and humans is consistent with an orally bioavailable, small-molecule drug. The compound (10 mM) was profiled in cryopreserved hepatocytes from five species: CD-1 mouse, Sprague–Dawley rat, beagle dog, cynomolgus monkey, and human. The peak areas from UV absorbance were used to calculate the percent distribution of each metabolite and then used to propose the metabolic pathway of RGN3067. The structures of the metabolites were elucidated based on the MS data. Calibration curves for the metabolites of RGN3067 were not established, and hence, the comparisons between species are qualitative. In addition to RGN3067, a total of 10 metabolites of RGN3067 were detected and identified in mouse, rat, dog, monkey, and human hepatocytes (Supplementary Table S2). In all species, the major products resulted from amide hydrolysis (M3, M4, M6) or *O*-demethylation/*O*-dealkylation and mono-oxygenation (M9/M10) (Supplementary Figure S1). However, the overall patterns of metabolism appear to be consistent between species, and the major metabolic pathways in human hepatocytes (*O*-demethylation, mono-oxygenation, and amide hydrolysis) were reproduced in mouse, rat, dog, and monkey hepatocytes (Supplementary Figure S1).

### 3.4. RGN3067 and Analogs Inhibit the Viability of Human Glioblastoma Cancer Cell Lines

**Figure 1:**
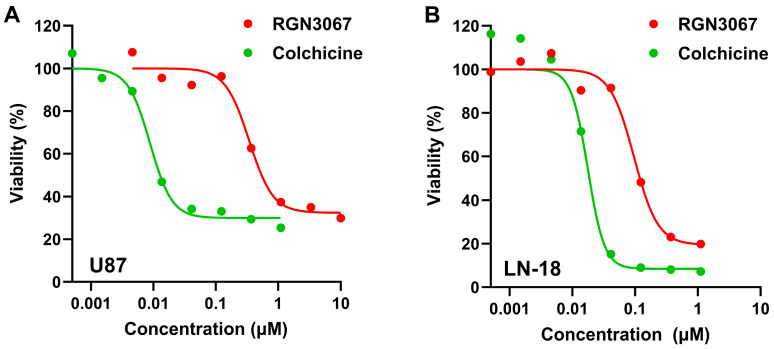
RGN3067 inhibits the viability of glioblastoma cell lines. (**A**,**B**) Representative dose–response curves of cell viability after treatment with RGN3067 and colchicine (positive control). U87 (**A**) and LN-18 (**B**) cells were exposed to compounds in dose response using an 8-point half-log dilution series (4.5 nM–10 µM). Cell viability was assessed after 72 h using the alamarBlue assay. Data were normalized to DMSO-treated cells. IC_50_ curves were fitted with a nonlinear regression model (Absolute IC_50_) using GraphPad v10. The mean absolute IC_50_ values from at least three independent experiments are shown in Supplementary Table S3.

Gliomas are particularly sensitive to MTAs, but most MTAs do not cross the BBB [4]. Since our compounds show a favorable profile for BBB penetration, we tested their ability to inhibit the growth of glioblastoma cells, including U87 cells and the TMZ-resistant cell line LN-18, in the alamarBlue cell viability assay. Treatment with RGN3067 for 72 h resulted in a strong inhibitory effect on cell viability, with average IC_50_ values of 560 nM (U87) and 117 nM (LN-18) (Figure 1A,B; Supplementary Table S3). Colchicine, which potently suppresses cell viability, was used as a positive control. Similar results were seen in other glioma lines (Supplementary Table S3), suggesting that RGN3067′s effects on cell growth are not cell-line-specific. Additionally, RGN3062 showed average IC_50_ values of 588 nM (U87) and 73 nM (LN-18), whereas the inactive isomer RGN3096 showed lower potency in these cell lines, with IC_50_ values of >10,000 nM (U87) and 2431 nM (LN-18) (Supplementary Figure S2A,B; Supplementary Table S3). Lastly, we showed that RGN3067 does not significantly inhibit the viability of the non-tumorigenic microglial cell line HMC3 (Supplementary Figure S2C).

### 3.5. RGN3067 and Analogs Inhibit Tubulin Polymerization and Induce Cell Cycle Arrest

**Figure 2:**
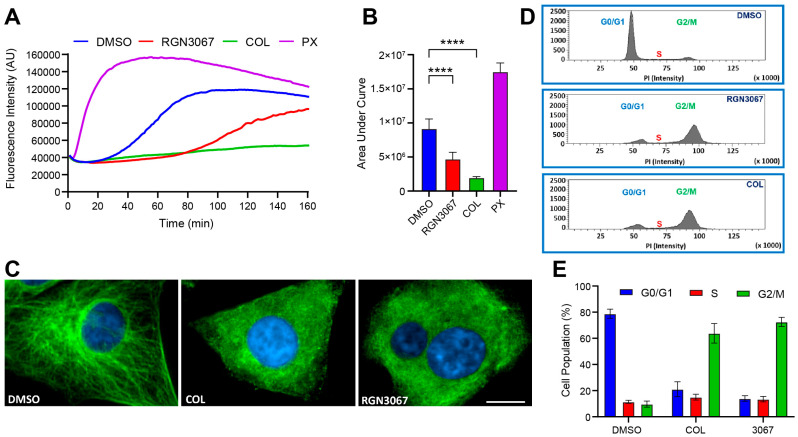
RGN3067 inhibits tubulin polymerization in vitro and triggers G2/M arrest in glioblastoma cells. (**A**,**B**) Representative tubulin polymerization plots and data quantification. DMSO, RGN3067 (5 μM), colchicine (5 μM), and paclitaxel (3 μM) were co-incubated with porcine tubulin (20 μM) in general tubulin buffer containing 15% glycerol and 1 mM GTP. Fluorescence was detected every min for 160 min (EX: 350/EM: 435) at 37 °C using a CLARIOstar microplate reader. **** *p* < 0.0001. (**C**) Immunofluorescence staining of β-tubulin in U87 glioblastoma cells. Cells were treated with DMSO, RGN3067 (0.5 μM), and colchicine (20 nM) and stained for β-tubulin-III (green) and the Hoechst 33342 (blue) nuclear marker. Images were captured using an Operetta confocal microscope at 40× magnification. Scale bar, 10 μm. (**D**) Representative histograms of cell cycle analysis of U87 cells treated with DMSO, RGN3067 (5 μM), and colchicine (20 nM) for 48 h and stained with propidium iodide (PI). Data was collected with a BD FACS Canto II Flow Cytometer and analyzed using the BD FACSDiva software. (**E**) The percentage of cells in G1, S, and G2/M phases from the histograms in (**D**). Data are plotted as mean ± SD from at least three independent experiments. Analysis between groups in (**B**) was performed using a one-way ANOVA with Tukey’s multiple comparisons test.

We assessed the ability of RGN3067 to reduce the polymerization of tubulin in vitro. In this assay, a fluorescent reporter is incorporated into polymerizing tubulin, resulting in a fluorescence readout of MT polymerization. The MT destabilizer colchicine (COL) and MT stabilizer paclitaxel (PX) were used as control MTAs. Area under the curve (AUC) values were used as a measure of microtubule formation and calculated from the tubulin polymerization curves shown in Figure 2A. Consistent with previous reports, COL robustly inhibited tubulin polymerization by 78%, whereas PX increased tubulin polymerization by 91%. RGN3067 showed a 49% reduction in tubulin polymerization (Figure 2A,B). Additionally, we tested the two isomers RGN3062 and RGN3096, which only differ in the spatial orientation of two hydrogens in their three-dimensional conformation. RGN3062 reduced tubulin polymerization by 36%, whereas its inactive isomer RGN3096 showed no difference relative to the DMSO control (Supplementary Figure S3A,B), indicating that the inhibition of tubulin polymerization by the compounds is unlikely to result from nonspecific binding.

We then examined the morphological effects of the compounds on the MT network using immunocytochemistry. U87 glioblastoma cells were treated with compounds at their IC_50_ concentrations for 24 h and stained for DNA and β-tubulin. As shown in Figure 2C, DMSO-treated U87 cells exhibited normal MT arrangement and organization. Cells treated with RGN3067 and COL displayed varying levels of disruption of tubulin organization, leading to defects in cell integrity. Cells lost their spindle shape and assumed a larger, flatter, and more rounded appearance due to the disruption of MT dynamics. Cells treated with RGN3062 also showed substantial tubulin disruption, whereas the inactive isomer RGN3096 had no visible effect on tubulin and cell structure (Supplementary Figure S3C).

Additionally, MTAs characteristically induce cell cycle arrest at the G2/M phase [11]. We investigated the effects of the compounds on cell cycle progression in U87 glioblastoma cells. After a 48 h treatment with RGN3067 (5 µM), 73% of cells were arrested at G2/M (Figure 2D,E). The G2/M arrest profile of RGN3067 was similar to that of colchicine (20 nM), which showed 64% of cells were G2/M phase-arrested (Figure 2D,E). Furthermore, U87 cells treated with the isomer RGN3062 showed 73% of cells arrested in G2/M, whereas only 11% of cells were arrested in G2/M with the inactive isomer RGN3096 at the same concentration (Supplementary Figure S3D,E). The inhibition of tubulin polymerization and G2/M cell cycle arrest is characteristic of compounds that target tubulin.

### 3.6. Compounds Bind to the Colchicine Binding Site of β-Tubulin

**Figure 3:**
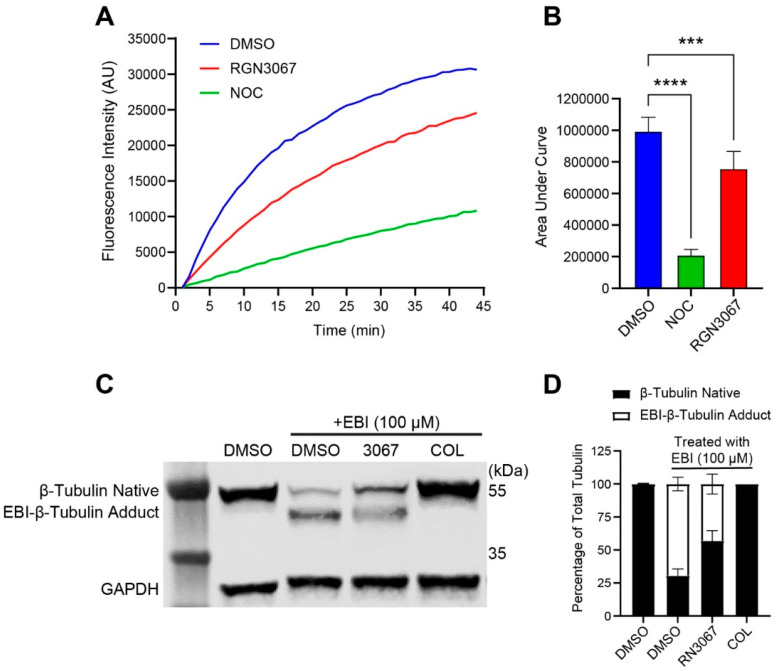
RGN3067 binds to the colchicine binding site in β-tubulin. (**A**) Fluorescence-based colchicine competitive binding assay. Porcine tubulin (20 μM) was incubated with DMSO, RGN3067 (50 µM), and the colchicine binding site inhibitor nocodazole (NOC, 50 µM) for 45 min at 37 °C in tubulin buffer containing 0.2 mM GTP, followed by incubation with colchicine (10 μM final) for 45 min. Fluorescence of the colchicine–tubulin complex was measured using a CLARIOstar Plus microplate reader (EX: 380 nm/EM: 435 nm). (**B**) Quantification of the fluorescence-based colchicine competitive binding assay. *** *p* < 0.001, **** *p* < 0.0001. (**C**) EBI competition assay in MCF-7 breast cancer cells. Cells were incubated with RGN3067 (50 µM) and colchicine (50 μM) for 2 h, followed by EBI (100 μM) for 2 h. Total proteins were lysed and subjected to Western blot analysis for β-tubulin. The β-tubulin adduct formed by EBI is detectable as a second immunoreacting band of β-tubulin. GAPDH was employed as the loading control. (**D**) Quantification of the Western blot in (**C**). The stacked bar graph shows the percentage of native β-tubulin (black) vs. EBI-β-tubulin adduct (white) of total tubulin in cells treated with compounds. Data are plotted as mean ± SD from at least three independent experiments. Analysis between groups in (**B**) was performed using a one-way ANOVA with Tukey’s multiple comparison test.

The colchicine binding site (CBS) of tubulin is one of the most important pockets for tubulin polymerization inhibitors [12]. We evaluated whether RGN3067 bound the CBS using a competitive binding assay that takes advantage of the fluorescence increase induced by COL upon binding to purified brain β-tubulin [13]. Preincubation with a compound that binds to the CBS interferes with COL-β-tubulin binding and reduces fluorescence. Nocodazole (NOC), a MT destabilizer that binds to the CBS, was used as a control. RGN3067 quenched fluorescence by 24% relative to the control, whereas NOC reduced fluorescence by 79% (Figure 3A,B). These data are consistent with RGN3067 binding to the CBS of β-tubulin.

In addition to the biochemical assay, we confirmed that our compounds bound to the colchicine binding pocket using the cellular N,N′-ethylene-bis (iodoacetamide) (EBI) competition assay [14]. EBI reacts with two conserved cysteine residues in the colchicine binding pocket of β-tubulin (amino acids 239 and 354), creating a more compact structure (EBI adduct) that runs faster than the native β-tubulin band on SDS-PAGE. Compounds that occupy the CBS inhibit the formation of the β-tubulin adduct. Because the U87 glioma cell line carries the β-tubulin III isoform that contains serine (Ser) in place of Cysteine (Cys) at position 239 (UniProt Q13509), we performed the assay with MCF-7 breast cancer cells, which contain β-tubulin isoforms bearing the requisite Cys residues [14]. We found that RGN3067 reduces the formation of the EBI–β-tubulin adduct (Figure 3C,D). These data are consistent with the antimitotic effects of our compounds resulting from binding to the CBS in β-tubulin.

### 3.7. The Effect of RGN3067 on Cell Viability Is Reversible

The reversibility of tubulin binding is a crucial parameter affecting the toxicity of tubulin inhibitors in vivo [15,16,17]. We evaluated the reversibility of RGN3067 in cells using a method based on that of Niu et al. [15]. U87 cells were treated with various concentrations of (1) RGN3067, (2) the nearly irreversible tubulin inhibitor colchicine [18,19], and (3) the investigational new drug sabizabulin (SAB), which binds to the colchicine pocket [20] and has a favorable safety profile based on preliminary antitumor activity in cancer models [21].

**Figure 4:**
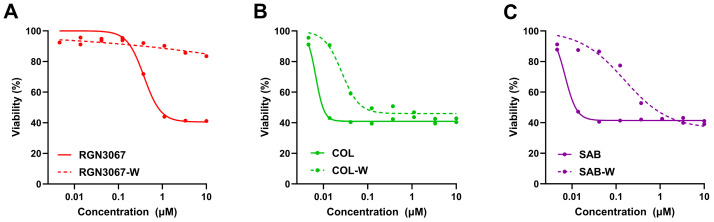
RGN3067 induces reversible effects on cell viability. (**A**–**C**) Representative IC_50_ curves for the reversibility experiments. U87 cells were treated with RGN3067 (**A**) and control compounds colchicine (COL, **B**), and sabizabulin (SAB, **C**) in dose response using an 8-point half-log dilution series (4.5 nM–10 µM). Compounds were washed out with medium after 6 h or allowed to remain in the wells. Cell viability was detected after 72 h with the alamarBlue assay. IC_50_ curves were fitted with a nonlinear regression model (Absolute IC_50_) using GraphPad v10. The mean absolute IC_50_ values from at least three independent experiments are shown in Supplementary Table S4.

Compounds were either washed out with medium after 6 h or left in the wells. Cell viability was assessed with CellTiter-Glo after 72 h, and IC_50_ values were calculated. Figure 4A–C show cell viability curves for each compound. In the no-washout condition, COL, SAB, and RGN3067 showed a strong inhibition of cell viability, with IC_50_ values of 13 nM, 11 nM, and 571 nM, respectively (Supplementary Table S4). After washout, COL and SAB showed IC_50_ values of 80 nM and 354 nM in the cell viability assay, whereas RGN3067 showed little effect on cell viability, with an IC_50_ value >10,000 nM (Supplementary Table S4). These data suggest that COL is likely an irreversible tubulin binder, as there was no substantial change in cell viability after compound washout. In contrast, SAB washout resulted in increased cell viability (higher IC_50_ value), suggesting that it is likely a reversible tubulin binder. Like SAB, which has an excellent clinical safety profile, the effects of RGN3067 on cell viability were largely reversible, even at the maximum concentration of 10 µM. These results indicate that RGN3067 likely dissociates quickly, which may lead to a substantially improved toxicity profile relative to COL.

### 3.8. RGN3067 Is Orally Bioavailable and Displays Good Brain Penetration

**Figure 5:**
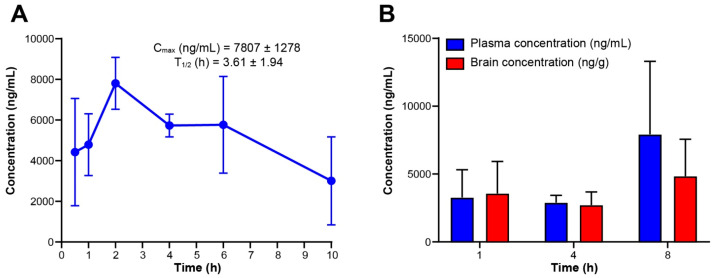
Pharmacokinetic evaluation of RGN3067. (**A**) Mean plasma concentration of RGN3067 after 100 mg/kg oral administration in normal CD-1 mice (*n* = 3). (**B**) Mean plasma and brain levels of RGN3067 with 100 mg/kg oral dose in CD-1 mice (*n* = 3). Data from (**A**,**B**) are shown as mean ± SD. Raw values are shown in Supplementary Tables S5 and S6.

Pharmacokinetic evaluation after oral administration of RGN3067 was determined in mice (CD1, male, fasting). Figure 5A shows the plasma levels of the compounds after an oral dose of 100 mg/kg of RGN3067. The compound reached a C_max_ of 7807 ng/mL (20 μM) with a T_max_ at 2 h and showed good exposure, as evidenced by the AUC value of 51,170 ng·h/mL (Supplementary Table S5). Representative LC-MS/MS chromatograms are shown in Supplementary Figure S4.

Because effective BBB penetration is essential for the treatment of GB, RGN3067 was evaluated for brain penetration in CD-1 mice at 1, 4 and 8 h after oral administration. RGN3067 shows equal distribution in plasma and brain at all time points (Figure 5B; Supplementary Table S6). These pharmacokinetic data validate our design approach and are also consistent with the favorable CNS MPO score. The MDR1-MDCK data are consistent with a lack of transport by the P-gp multidrug resistance protein and with passive diffusion across cellular membrane barriers, making RGN3067 a promising molecule for the treatment of brain and other CNS cancers protected by the BBB. Representative LC-MS/MS chromatograms are shown in Supplementary Figures S5 and S6.

### 3.9. RGN3067 Is Well Tolerated in Mice

**Figure 6:**
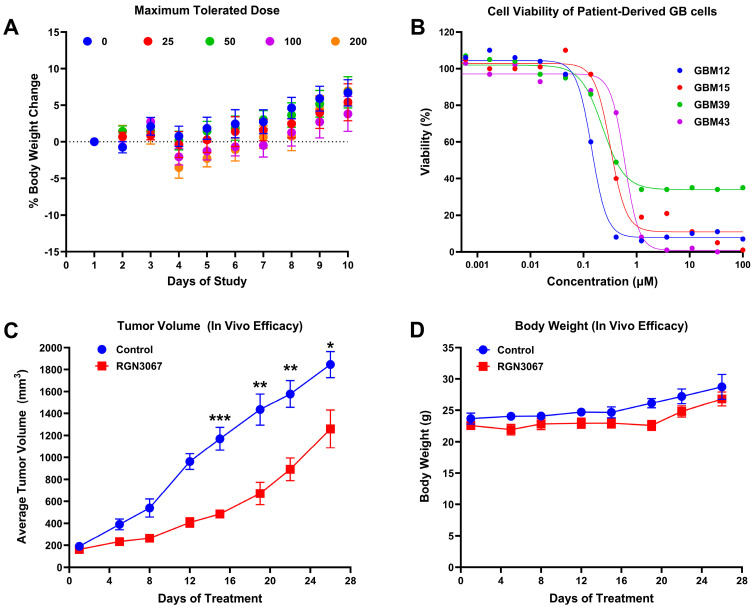
Effect of RGN3067 on a SC GBM12 xenograft model in mice. (**A**) MTD determination of doses up to 200 mg/kg of RGN3067 twice daily for 5 days + post drug for 5 days (*n* = 5). (**B**) RGN3067 shows activity against the PDX HGG cell lines GBM12, GBM15, GBM39, and GBM43 after a 144 h incubation with the compound. RGN3067 was tested using a 13-point half-log dilution series (0.2 nM–100 µM). (**C**,**D**) Mice (4–6 weeks old, n = 8 for treated vs. non-treated) were inoculated with patient-derived GBM12 cells, and tumors were allowed to grow to a volume of 150–250 mm^3^. Mice were then treated with 100 mg/kg of RGN3067 BID for 5 days, followed by 2 days of no treatment, for 4 cycles. Mice were euthanized when the tumor volume reached 2000 mm^3^. (**C**) Tumor sizes were measured twice per week until tumors were harvested. (**D**) No significant toxicity or weight loss was observed. Data in (**A**,**C**,**D**) are shown as mean ± SEM. Multiple unpaired *t*-tests were used in (**C**), and statistical significance was determined using the Holm–Šídák method. * *p* < 0.05; ** *p* < 0.01; *** *p* < 0.001.

To assess the tolerability of RGN3067 in vivo and to determine the levels of dosing appropriate for an efficacy study, we conducted a maximum tolerated dose study in athymic nude female mice. The same strain was used for PDX efficacy studies. Mice received oral doses of vehicle or RGN3067 at 25, 50, 100 or 200 mg/kg twice daily for 5 days, followed by observation for 5 days. No mice died during this study, and no statistically significant weight loss was observed (Figure 6A). A nonsignificant trend of weight loss was detected at the highest doses. No obvious changes in behavior or appearance (hair and skin) were observed. The dose of 100 mg/kg twice per day was taken as the maximum dose for subsequent studies due to solubility limitations.

### 3.10. RGN3067 Suppresses Tumor Growth in a GB PDX Model

RGN3067 was selected for in vivo evaluation based on its oral availability, promising pharmacokinetics, excellent BBB penetration, and potency against four patient-derived GB cell lines from the Brain Tumor PDX National Resource [22]: GBM12, GBM15, GBM39, and GBM43. All patient-derived cell lines were sensitive to RGN3067, particularly GBM12, with an IC_50_ of 148 nM (Figure 6B; Supplementary Table S3).

Hence, efficacy was tested in a subcutaneous (SC) mouse model of glioblastoma using GBM12 patient-derived cells for tumor generation. An amount of 100 mg/kg of RGN3067 was administered orally twice per day. The tumor volume was measured every three to four days during the four cycles of treatment. A statistically significant inhibition of tumor growth was observed beginning on day 14 of treatment and continuing through the end of the study (Figure 6C). Between cycle 1 and cycle 2, one animal was censored (deceased) from both the control and the treatment cohorts. Drug-related complications were rejected as the cause due to the parallel weight maintenance of both control and treated cohorts over the duration of the study. Athymic mice are vulnerable to infection or may succumb to repeated esophageal irritation/tearing from the oral gavage administration of a vehicle or drug. No changes in behavior or appearance (hair and skin) or statistically significant weight loss was observed (Figure 6D).

## 4. Discussion

Despite the development of new treatments for GB over the last few decades, the disease continues to have a poor prognosis, and these therapies have not impacted the median survival of 15 months after diagnosis [23]. Thus, innovative therapeutic strategies are urgently needed to delay recurrence and extend the survival of patients with GB tumors. Evidence suggests that gliomas are particularly sensitive to MTAs, and preclinical studies by this group and others have shown that tubulin-targeting therapies can be effective in treating brain cancers. Many MTAs, such as taxanes, vinca alkaloids, and epothilones, show potent anticancer activities in preclinical studies and clinical trials [4,24], but their effectiveness is often limited by poor aqueous solubility, multidrug resistance, inability to cross the BBB, and drug-induced cytotoxicity, such as peripheral neuropathy [25,26,27,28,29]. Besides the taxane and vinca alkaloid binding sites, the colchicine binding site of tubulin has been examined extensively. While colchicine itself has had limited clinical success due to its high toxicity and low therapeutic index [30], colchicine site inhibitors are attractive molecules due to several advantages they possess over taxane- and vinca-binding site compounds, including selective toxicity against tumor vasculature and an insensitivity to P-gp efflux pump-mediated multidrug resistance [31,32,33].

This study describes the development of RGN3067, a MTA that was designed to cross the BBB using CNS MPO parameters. We showed that RGN3067 inhibits the polymerization of tubulin in vitro and binds to the colchicine binding pocket of β-tubulin. In line with other tubulin destabilizers, we found that RGN3067 induces cell cycle arrest in the G2/M phase and inhibits the growth of human GB cancer cell lines (including the TMZ-resistant cell line LN-18) and patient-derived GB cell lines, with nanomolar potency. The effectiveness of RGN3067 in the TMZ-resistant line LN-18 is worth noting, as TMZ resistance remains a significant barrier to the successful treatment of patients with HGG [34]. Only 50% percent of GB patients are responsive to TMZ, and the vast majority of responsive patients eventually develop TMZ resistance. This resistance is most commonly due to the overexpression of O6-methylguanine methyltransferase (MGMT), which is frequently correlated with the loss of epigenetic silencing of the *MGMT* promoter. LN-18 cells replicate the loss of epigenetic silencing and show constitutively high levels of MGMT activity [35]. Due to the significance of TMZ resistance in the clinic, future in vivo efficacy studies will include TMZ-resistant tumors.

RGN3067 penetrates the rodent brain effectively, showing that a favorable CNS MPO score and the lack of P-gp pump efflux in the MDR1-MDCK model are accurate predictors of BBB penetrance. Equal amounts of RGN3067 were observed in the brain and plasma after oral administration. Furthermore, RGN3067 decreased the rate of tumor growth in a GB PDX model relative to control and caused no discernible toxicities in mice. A contributing factor to the low toxicity of RGN3067 in the PDX model may be its reversible binding to tubulin. Reversibility experiments showed that RGN3067 has a similar profile to sabizabulin, a potent tubulin destabilizer with a good safety profile in patients [20]. However, unlike RGN3067, SAB is not BBB-penetrant with a brain-to-plasma ratio between 5% and 9% in mice [36]. In contrast, the activity of colchicine, a classical MTA with a narrow therapeutic window, was nearly irreversible. These findings are notable from a therapeutic standpoint, as the low toxicity of RGN3067 makes it a promising candidate for the treatment of GB.

To facilitate the preclinical development of RGN3067, it was extensively profiled in eADME assays. The solubility and plasma binding of RGN3067 predict good exposure in vivo. The observed protein binding in human plasma is similar to that observed in rodents, and the human liver microsomal stability exceeds the stability observed in rodents. Metabolite ID studies in hepatocytes from multiple species indicate a consistent pattern of metabolism, increasing the likelihood that future animal studies will accurately predict human exposure and risk levels in the clinic for this class of molecules.

In summary, the design of RGN3067 addresses the limitations in physicochemical properties that have prevented previous MTAs from clinical success for the treatment of GB and other brain cancers. Our family of small-molecule tubulin destabilizers have been optimized chemically to cross the BBB, with the goal of developing a new generation of MTAs to treat glioblastoma and other CNS cancers. We show that our proof-of-concept molecules penetrate the brains of mice and have activity against glioma cells both in vitro and in a GB PDX model.

## 5. Conclusions

Our study demonstrates that the small-molecule tubulin destabilizer RGN3067 is orally bioavailable and BBB-penetrant. It potently reduces the viability of GB cell lines and patient-derived, low-passage, genetically diverse GB cells in vitro. Although RGN3067 does not lead to tumor eradication, it suppresses the growth of patient-derived GB cells in an SC murine model. Further development and characterization of RGN3067 analogs may lead to treatments for GB and other types of brain cancer.

## Figures and Tables

**Table 1:** Structures of compounds used in the study.

Compound	Structure	Isomer	Molecular Weight	Calculated log D	CNS MPO Score
RGN3067	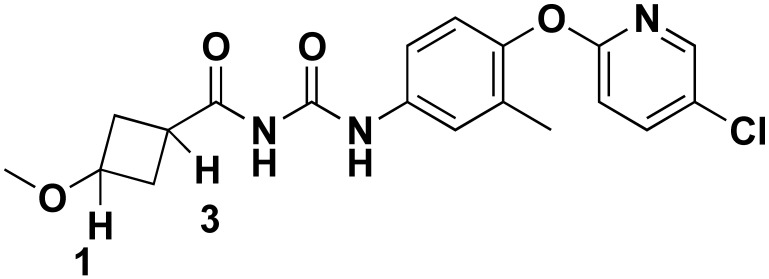	cis	389.8	2.25	5.00
RGN3062	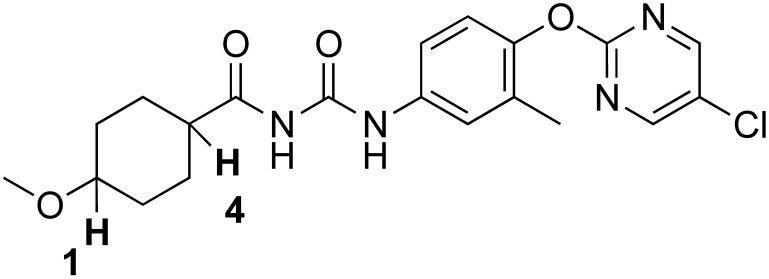	cis	418.8	3.1	4.1
RGN3096	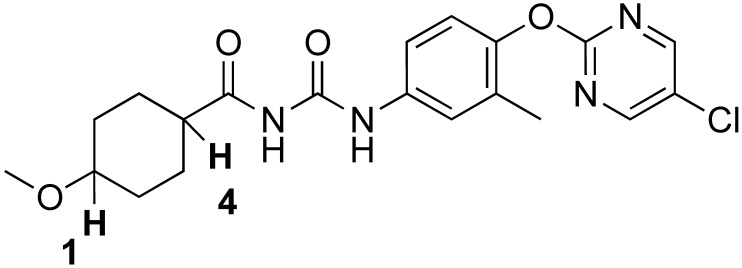	trans	418.8	3.1	4.1

## Data Availability

Data is contained within the article or Supplementary Materials.

## Supplementary Materials

The following supporting information can be downloaded at: https://www.mdpi.com/article/10.3390/biomedicines12020406/s1, Figure S1: Proposed clearance pathways of RGN3067 in hepatocytes; Figure S2: Effects of RGN compounds on the cell viability of glioblastoma and non-tumorigenic microglial cells; Figure S3: Isomers RGN3062 and RGN3096 inhibit tubulin polymerization and trigger G2/M arrest; Figure S4: Representative LC-MS/MS chromatograms for the pharmacokinetic study on RGN3067 at the 30 min time point (mouse plasma); Figure S5: Representative LC-MS/MS chromatograms for the brain permeability study on RGN3067 at 1 and 8 h time points (mouse plasma); Figure S6: Representative LC-MS/MS chromatograms for the brain permeability study on RGN3067 at 1 and 8 h time points (mouse brain); Table S1: eADME profile of RGN3067; Table S2: Metabolite identification of RGN3067; Table S3: RGN compounds are active in glioblastoma cell lines; Table S4: Reversibility experiments with RGN3067; Table S5: Pharmacokinetics of oral administration of RGN3067 in CD-1 mice; Table S6: Plasma and brain levels of RGN3067 after a 100 mg/kg oral dose in CD-1 mice.

## References

[1] Ostrom Q.T., Patil N., Cioffi G., Waite K., Kruchko C., Barnholtz-Sloan J.S. (2020). CBTRUS Statistical Report: Primary Brain and Other Central Nervous System Tumors Diagnosed in the United States in 2013–2017. Neuro-Oncology.

[2] Seystahl K., Wick W., Weller M. (2016). Therapeutic options in recurrent glioblastoma—An update. Crit. Rev. Oncol./Hematol..

[3] Christensen S.B. (2022). Drugs That Changed Society: Microtubule-Targeting Agents Belonging to Taxanoids, Macrolides and Non-Ribosomal Peptides. Molecules.

[4] Calinescu A.-A., Castro M.G. (2016). Microtubule targeting agents in glioma. Transl. Cancer Res..

[5] Jung E., Alfonso J., Monyer H., Wick W., Winkler F. (2020). Neuronal signatures in cancer. Int. J. Cancer.

[6] Duly A.M.P., Kao F.C.L., Teo W.S., Kavallaris M. (2022). *βIII-Tubulin* Gene Regulation in Health and Disease. Front. Cell Dev. Biol..

[7] Das T., Anand U., Pandey S.K., Ashby C.R., Assaraf Y.G., Chen Z.S., Dey A. (2021). Therapeutic strategies to overcome taxane resistance in cancer. Drug Resist. Updates.

[8] Sun K., Sun Z., Zhao F., Shan G., Meng Q. (2021). Recent advances in research of colchicine binding site inhibitors and their interaction modes with tubulin. Future Med. Chem..

[9] Wager T.T., Hou X., Verhoest P.R., Villalobos A. (2016). Central Nervous System Multiparameter Optimization Desirability: Application in Drug Discovery. ACS Chem. Neurosci..

[10] Tomayko M.M., Reynolds C.P. (1989). Determination of subcutaneous tumor size in athymic (nude) mice. Cancer Chemother. Pharmacol..

[11] Bates D., Eastman A. (2017). Microtubule destabilising agents: Far more than just antimitotic anticancer drugs. Br. J. Clin. Pharmacol..

[12] Steinmetz M.O., Prota A.E. (2018). Microtubule-Targeting Agents: Strategies to Hijack the Cytoskeleton. Trends Cell Biol..

[13] Bhattacharyya B., Wolff J. (1974). Promotion of fluorescence upon binding of colchicine to tubulin. Proc. Natl. Acad. Sci. USA.

[14] Fortin S., Lacroix J., Côté M.-F., Moreau E., Petitclerc É., C-Gaudreault R. (2010). Quick and simple detection technique to assess the binding of antimicrotubule agents to the colchicine-binding site. Biol. Proced. Online.

[15] Niu L., Yang J., Yan W., Yu Y., Zheng Y., Ye H., Chen Q., Chen L. (2019). Reversible binding of the anticancer drug KXO1 (tirbanibulin) to the colchicine-binding site of beta-tubulin explains KXO1’s low clinical toxicity. J. Biol. Chem..

[16] Thomas N.E., Thamkachy R., Sivakumar K.C., Sreedevi K.J., Louis X.L., Thomas S.A., Kumar R., Rajasekharan K.N., Cassimeris L., Sengupta S. (2014). Reversible action of diaminothiazoles in cancer cells is implicated by the induction of a fast conformational change of tubulin and suppression of microtubule dynamics. Mol. Cancer Ther..

[17] Yan W., Yang T., Yang J., Wang T., Yu Y., Wang Y., Chen Q., Bai P., Li D., Ye H. (2018). SKLB060 Reversibly Binds to Colchicine Site of Tubulin and Possesses Efficacy in Multidrug-Resistant Cell Lines. Cell. Physiol. Biochem..

[18] Banerjee A.C., Bhattacharyya B. (1979). Colcemid and colchicine binding to tubulin. Similarity and dissimilarity. FEBS Lett..

[19] Towle M.J., Salvato K.A., Wels B.F., Aalfs K.K., Zheng W., Seletsky B.M., Zhu X., Lewis B.M., Kishi Y., Yu M.J. (2011). Eribulin induces irreversible mitotic blockade: Implications of cell-based pharmacodynamics for in vivo efficacy under intermittent dosing conditions. Cancer Res..

[20] Markowski M.C., Tutrone R., Pieczonka C., Barnette K.G., Getzenberg R.H., Rodriguez D., Steiner M.S., Saltzstein D.R., Eisenberger M.A., Antonarakis E.S. (2022). A Phase Ib/II Study of Sabizabulin, a Novel Oral Cytoskeleton Disruptor, in Men with Metastatic Castration-resistant Prostate Cancer with Progression on an Androgen Receptor-targeting Agent. Clin. Cancer Res..

[21] Krutilina R.I., Hartman K.L., Oluwalana D., Playa H.C., Parke D.N., Chen H., Miller D.D., Li W., Seagroves T.N. (2022). Sabizabulin, a Potent Orally Bioavailable Colchicine Binding Site Agent, Suppresses HER2+ Breast Cancer and Metastasis. Cancers.

[22] Vaubel R.A., Tian S., Remonde D., Schroeder M.A., Mladek A.C., Kitange G.J., Caron A., Kollmeyer T.M., Grove R., Peng S. (2020). Genomic and Phenotypic Characterization of a Broad Panel of Patient-Derived Xenografts Reflects the Diversity of Glioblastoma. Clin. Cancer Res..

[23] Stupp R., Hegi M.E., Mason W.P., van den Bent M.J., Taphoorn M.J.B., Janzer R.C., Ludwin S.K., Allgeier A., Fisher B., Belanger K. (2009). Effects of radiotherapy with concomitant and adjuvant temozolomide versus radiotherapy alone on survival in glioblastoma in a randomised phase III study: 5-year analysis of the EORTC-NCIC trial. Lancet Oncol..

[24] Čermák V., Dostál V., Jelínek M., Libusová L., Kovář J., Rösel D., Brábek J. (2020). Microtubule-targeting agents and their impact on cancer treatment. Eur. J. Cell Biol..

[25] Fellner S., Bauer B., Miller D.S., Schaffrik M., Fankhänel M., Spruss T., Bernhardt G., Graeff C., Färber L., Gschaidmeier H. (2002). Transport of paclitaxel (Taxol) across the blood-brain barrier in vitro and in vivo. J. Clin. Investig..

[26] Greig N.H., Soncrant T.T., Shetty H.U., Momma S., Smith Q.R., Rapoport S.I. (1990). Brain uptake and anticancer activities of vincristine and vinblastine are restricted by their low cerebrovascular permeability and binding to plasma constituents in rat. Cancer Chemother. Pharmacol..

[27] Krause W. (2019). Resistance to anti-tubulin agents: From vinca alkaloids to epothilones. Cancer Drug Resist..

[28] Velasco R., Bruna J. (2015). Taxane-Induced Peripheral Neurotoxicity. Toxics.

[29] Wu X., Wang Q., Li W. (2016). Recent Advances in Heterocyclic Tubulin Inhibitors Targeting the Colchicine Binding Site. Anti-Cancer Agents Med. Chem..

[30] Ghawanmeh A.A., Chong K.F., Sarkar S.M., Abu Bakar M., Othaman R., Khalid R.M. (2018). Colchicine prodrugs and codrugs: Chemistry and bioactivities. Eur. J. Med. Chem..

[31] Gangjee A., Zhao Y., Lin L., Raghavan S., Roberts E.G., Risinger A.L., Hamel E., Mooberry S.L. (2010). Synthesis and discovery of water-soluble microtubule targeting agents that bind to the colchicine site on tubulin and circumvent Pgp mediated resistance. J. Med. Chem..

[32] Ji Y.-T., Liu Y.-N., Liu Z.-P. (2015). Tubulin colchicine binding site inhibitors as vascular disrupting agents in clinical developments. Curr. Med. Chem..

[33] Lu Y., Chen J., Xiao M., Li W., Miller D.D. (2012). An overview of tubulin inhibitors that interact with the colchicine binding site. Pharm. Res..

[34] Lee S.Y. (2016). Temozolomide resistance in glioblastoma multiforme. Genes Dis..

[35] Hegi M.E., Liu L., Herman J.G., Stupp R., Wick W., Weller M., Mehta M.P., Gilbert M.R. (2008). Correlation of *O^6^-methylguanine methyltransferase (MGMT)* promoter methylation with clinical outcomes in glioblastoma and clinical strategies to modulate MGMT activity. J. Clin. Oncol..

[36] Li C.-M., Lu Y., Chen J., Costello T.A., Narayanan R., Dalton M.N., Snyder L.M., Ahn S., Li W., Miller D.D. (2012). Orally bioavailable tubulin antagonists for paclitaxel-refractory cancer. Pharm. Res..

