# Supplementary material for: Novel Brain-Penetrant, Small-Molecule Tubulin Destabilizers for the Treatment of Glioblastoma
Source: Biomedicines. 2024 Feb 9;12(2):406. doi: 10.3390/biomedicines12020406 (PMC10887108; doi:10.3390/biomedicines12020406)
Supplement: Supplementary file 1 [file biomedicines-12-00406-s001.zip › biomedicines-2813006-supplementary.pdf]

SUPPLEMENTARY MATERIALS

Synthetic Scheme for RGN3062 and RGN3096

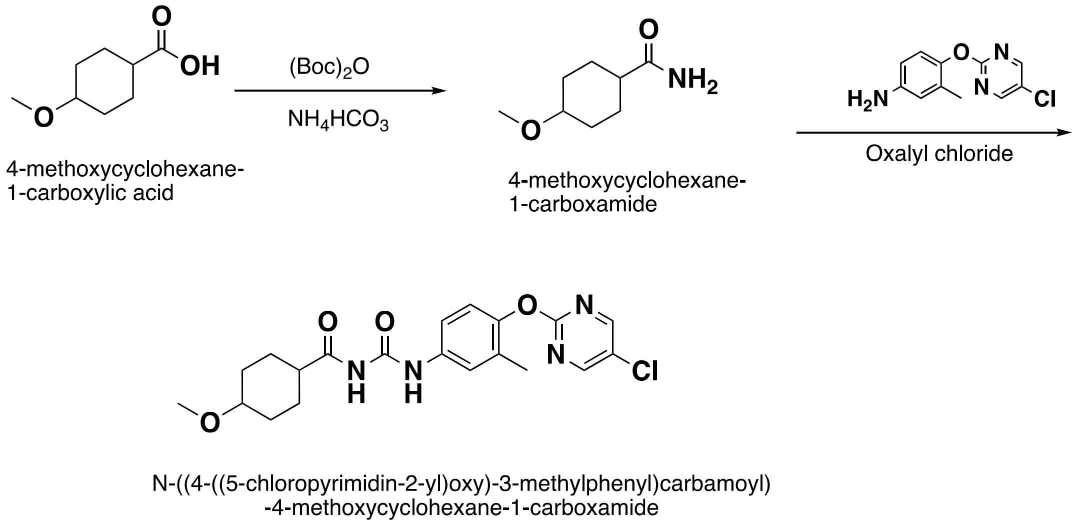

RGN3062 and RGN3096

4-((5-chloropyrimidin-2-yl)oxy)-3-methylaniline. A solution of 4-amino-2-methylphenol (830.0 mg, 6.71 mmol), 2,5-dichloropyrimidine (1.00 g, 6.71 mmol), potassium carbonate (2.80 g, 20.13 mmol) in *N,N*-dimethylformamide (20 mL) was stirred at 100 °C for 16 h. After quenching the reaction, the reaction mixture was extracted with ethyl acetate (50 mL × 3). The organic layer was washed with brine (100 mL), dried over Na_2SO_4, and concentrated. The crude product was purified by flash column chromatography (petroleum ether: methyl t-butyl ether = 11: 9) to get 4-((5-chloropyrimidin-2-yl)oxy)-3-methylaniline (1.20 g) (yield 75.9%) as a brown solid. LC-MS (ESI$^+$): *m/z* 236.1 (M + H)$^+$.

4-Methoxycyclohexane-1-carboxamide

4-Methoxycyclohexane-1-carboxylic acid (500 mg, 3.16 mmol), pyridine (0.7 mL, 8.34 mmol), di-tert-butyl dicarbonate (758 mg, 3.48 mmol) and ammonium bicarbonate (375 mg, 4.74 mmol) were added to freshly distilled tetrahydrofuran (20 mL). The reaction mixture was stirred at room temperature for 16 h, then petroleum ether was added, and the precipitate was filtered. The filter cake was dried to afford 4-methoxycyclohexane-1-carboxamide (380 mg) (Yield 76%) as a white solid.

N-((4-((5-chloropyrimidin-2-yl)oxy)-3-methylphenyl)carbamoyl)-4-methoxycyclohexane-1-carboxamide

To a solution of 4-methoxycyclohexane-1-carboxamide (200 mg, 1.27 mmol) in 1, 2-dichloroethane (8 mL) at room temperature was added oxalyl chloride (242 mg, 1.91 mmol). The reaction mixture was heated at 80 °C for 6 h, and then concentrated. The resulting mixture was dissolved in 1, 2-dichloroethane (8 mL), the resultant solution was cooled to 0 °C and then treated with 4-((5-chloropyrimidin-2-yl)oxy)-3-methylaniline (200 mg, 0.85 mmol). The mixture was allowed to warm up to room temperature and stirred for 16 h. The mixture was concentrated, and the residue was purified by HPLC to afford RGN3062 and RGN3096 as a white solid.

RGN3062

LC-MS (ESI^+): *m/z* 419 $(M + H)^+$.

^1H NMR (400 MHz, DMSO-*d*6) *δ* 10.64 (s, 1H), 10.58 (s, 1H), 8.74 (s, 2H), 7.47–7.42 (m, 2H), 7.10–7.08 (m, 1H), 3.38–3.35 (m, 1H), 3.21(s, 3H), 2.50–2.47 (m, 1H), 2.05 (s, 3H), 1.89–1.85 (m, 2H), 1.69-1.62 (m, 2H), 1.58–1.52 (m, 2H), 1.42–1.35 (m, 2H).

HPLC Purity = 100 % by UV 254 nm.

RGN3096

LC-MS (ESI^+): *m/z* 419 $(M + H)^+$.

^1H NMR (400 MHz, DMSO-*d*6) *δ* 10.64 (s, 1H), 10.54 (s, 1H), 8.72 (s, 2H), 7.44–7.40 (m, 2H), 7.09–7.07 (m, 1H), 3.22 (s, 3H), 3.09–3.07 (m, 1H), 2.48–2.36 (m, 1H), 2.06 (s, 3H), 1.88–1.85 (m, 2H), 1.47–1.42 (m, 2H), 1.39–1.36 (m, 2H), 1.28–1.22 (m, 2H).

HPLC Purity = 100 % by UV 254 nm.

Synthetic Scheme for RGN3067

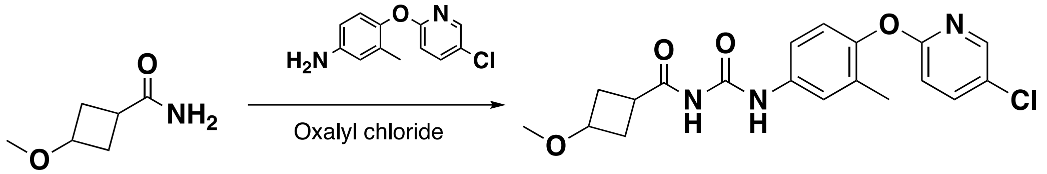

3-methoxycyclobutane-1-carboxamide

N-((4-((5-chloropyridin-2-yl)oxy)-3-methylphenyl)carbamoyl)-3-methoxycyclobutane-1-carboxamide

RGN3067

4-((5-chloropyridin-2-yl)oxy)-3-methylaniline. A solution of 4-amino-2-methylphenol (3.72 g, 30.21 mmol), 2,5-dichloropyridine (4.5 g, 30.21 mmol), potassium carbonate (12.5 g, 90.63 mmol) in DMF (60 mL) was stirred at 100 °C for 16 h under nitrogen. After quenching the reaction, the reaction mixture was extracted with ethyl acetate (300 mL × 3). The organic layer was washed with brine (300 mL), dried over Na_2SO_4, filtered, and concentrated. The crude product was purified by flash column chromatography (petroleum ether: ethyl acetate = 2: 1) to get 4-((5-chloropyridin-2-yl)oxy)-3-methylaniline (5.0 g) (Yield 70.2%) as a brown oily liquid. LC-MS (ESI^+): *m/z* 236.07 $(M + H)^+$.

N-((4-((5-chloropyridin-2-yl)oxy)-3-methylphenyl)carbamoyl)-3-methoxycyclobutane-1-carboxamide

To a solution of 3-methoxycyclobutane-1-carboxamide (100 mg, 0.7 mmol) in 1, 2-dichloroethane (8 mL) at room temperature was added oxalyl chloride (242 mg, 1.9 mmol). The reaction mixture was heated at 80 °C for 6 h, and then concentrated. The resulting mixture was dissolved in 1, 2-dichloroethane (8 mL), the resultant solution was cooled to 0 °C and then treated with 4-((5-chloropyridin-2-yl)oxy)-3-methylaniline (150 mg, 0.64 mmol) at 0 °C under N_2. The mixture was allowed to warm up to room temperature and stirred for 16 h. The mixture was concentrated, and the residue was purified by high performance liquid chromatography to afford RGN3067 (25.2 mg, 0.06 mmol) as a white solid.

LC-MS (ESI$^+$): *m/z* 390 (M + H)$^+$.

^{1}H NMR (400 MHz, DMSO-*d*6) δ 10.67 (s, 1H), 10.53 (s, 1H), 8.16 (d, *J* = 2.6 Hz, 1H), 7.95 (dd, *J* = 8.8, 2.7 Hz, 1H), 7.50–7.34 (m, 2H), 7.05 (dd, *J* = 16.5, 8.4 Hz, 2H), 3.84–3.74 (m, 1H), 3.14 (s, 3H), 2.81 (dd, *J* = 16.8, 8.4 Hz, 1H), 2.47-2.36 (m, 2H), 2.11–1.93 (m, 5H).

HPLC Purity = 100 % by UV 254 nm.

Details of LC-MS/MS method used for the analysis.

Instrument Triple Quad 6500+

Internal standard(s): 100 ng/mL Labetalol & 100 ng/mL Tolbutamide in acetonitrile

MS conditions ESI: positive

SRM detection

RGN3067 [M+H]+ m/z 390.1>261.1

Tolbutamide (Internal Standard) [M+H]+ m/z 271.1>155.3

Ultra-Performance Liquid Chromatography Conditions

Mobile Phase A: 0.1% Formic Acid in Water

Mobile Phase B: 0.1% Formic Acid in acetonitrile

Column: Waters ACQUITY UPLC BEH C18 2.1*50mm, 1.7μM

Flow rate: 0.6000 mL/min

Retention time:
RGN3067 1.48 min
Tolbutamide (IS) 1.34 min

Details of LC-MS/MS method used for the analysis in brain permeability study.

Instrument Triple Quad 6500+

Internal standard(s): 100 ng/mL Labetalol & 100 ng/mL Tolbutamide in acetonitrile

MS conditions ESI: positive
SRM detection
RGN3067 [M+H]+ m/z 390.1>261.1
Tolbutamide (IS) [M+H]+ m/z 271.1>155.3

Ultra-Performance Liquid Chromatography Conditions

Mobile Phase A: 0.1% Formic Acid in water
Mobile Phase B: 0.1% Formic Acid in acetonitrile

Column: Waters ACQUITY UPLC BEH C18 2.1*50mm, 1.7µM

Flow rate: 0.6000 mL/min
Retention time:

RGN3067	1.46 min
Tolbutamide (IS)	1.31 min

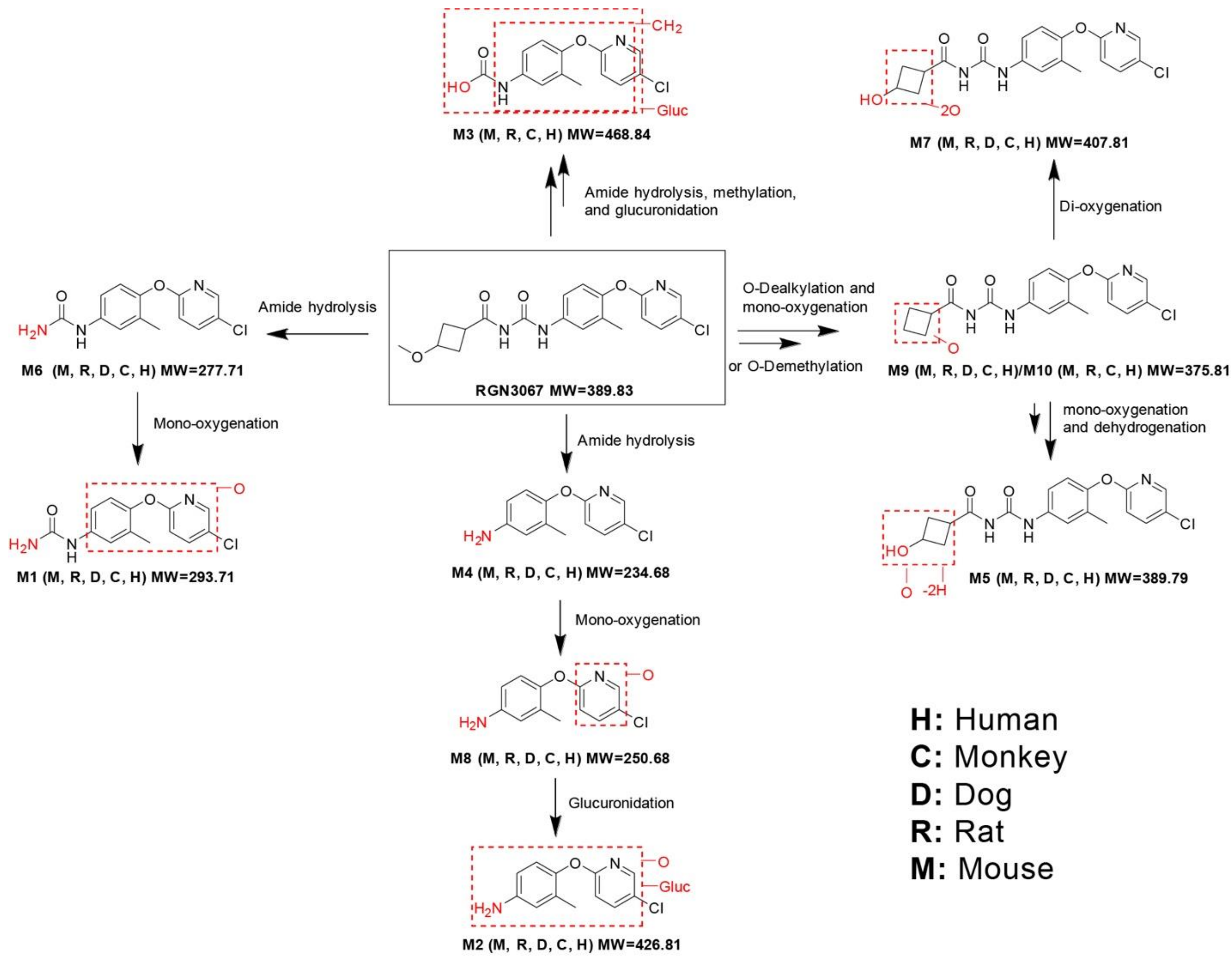

Figure S1. Proposed clearance pathways of RGN3067 in hepatocytes.

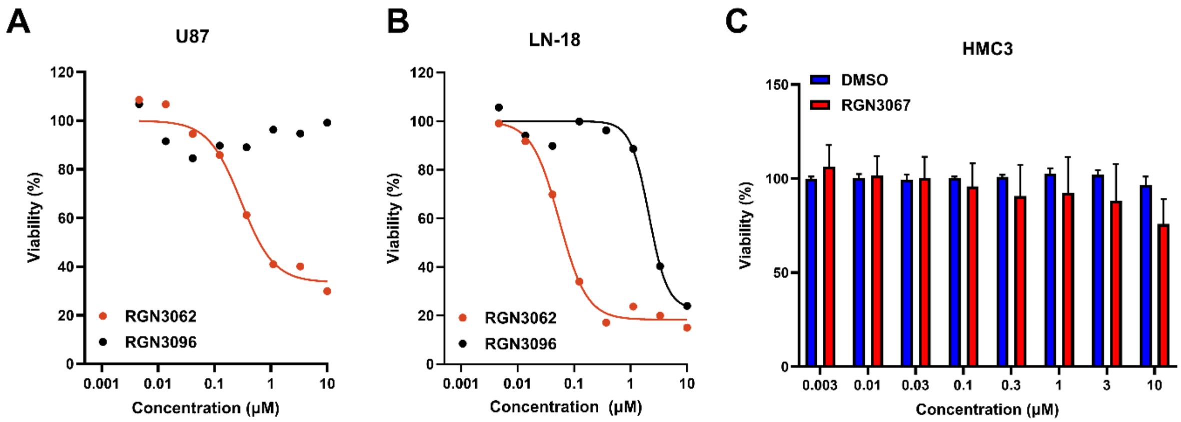

Figure S2. Effects of RGN compounds on the cell viability of glioblastoma and non-tumorigenic microglial cells. (A–B) Representative dose response curves showing cell viability of U87 (A) and LN-18 (B) cells after a 72 h exposure to RGN3062 and RGN3096. The mean absolute IC_{50} values are shown in Supplementary Table S3. (C) DMSO (control) and RGN3067 were tested in dose-response in the non-tumorigenic human microglial cell line HMC3. The viability of HMC3 cells was not significantly inhibited at RGN3067 concentrations up to 10 µM. Data are plotted as mean ± SD from at least three independent experiments.

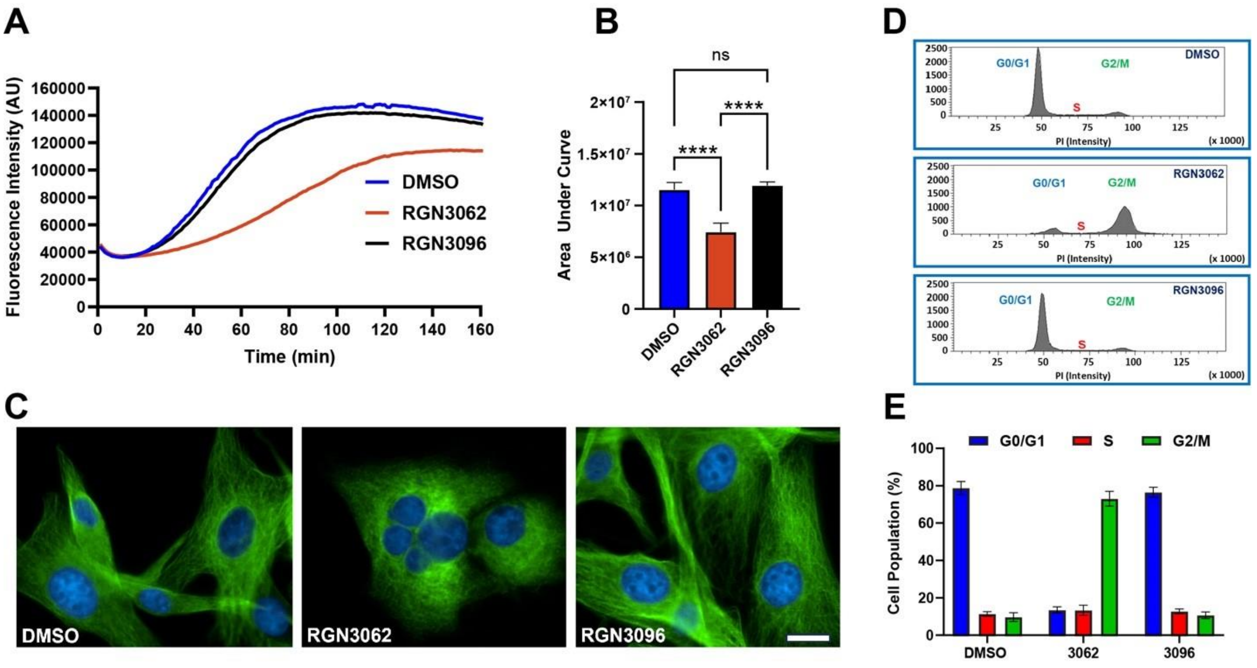

Figure S3. Isomers RGN3062 and RGN3096 inhibit tubulin polymerization and trigger G2/M arrest. (A–B) Representative plots and quantification of DMSO, RGN3062 (5 μM), and RGN3096 (5 μM) in the tubulin polymerization assay. (C) Immunofluorescence staining of β-tubulin in U87 glioblastoma cells treated with DMSO, RGN3067 (1 μM), and RGN3096 (1 μM). Scale bar, 10 μm. (D) Representative histograms of cell cycle analysis of U87 cells treated with DMSO, RGN3062 (5 μM), and RGN3096 (5 μM) for 48 h. (E) The percentage of cells in G1, S, and G2/M phases from the histograms in (D). Data are plotted as mean ± SD from at least three independent experiments. Analysis between groups in (B) was performed using a one-way ANOVA with Tukey's multiple comparisons test.

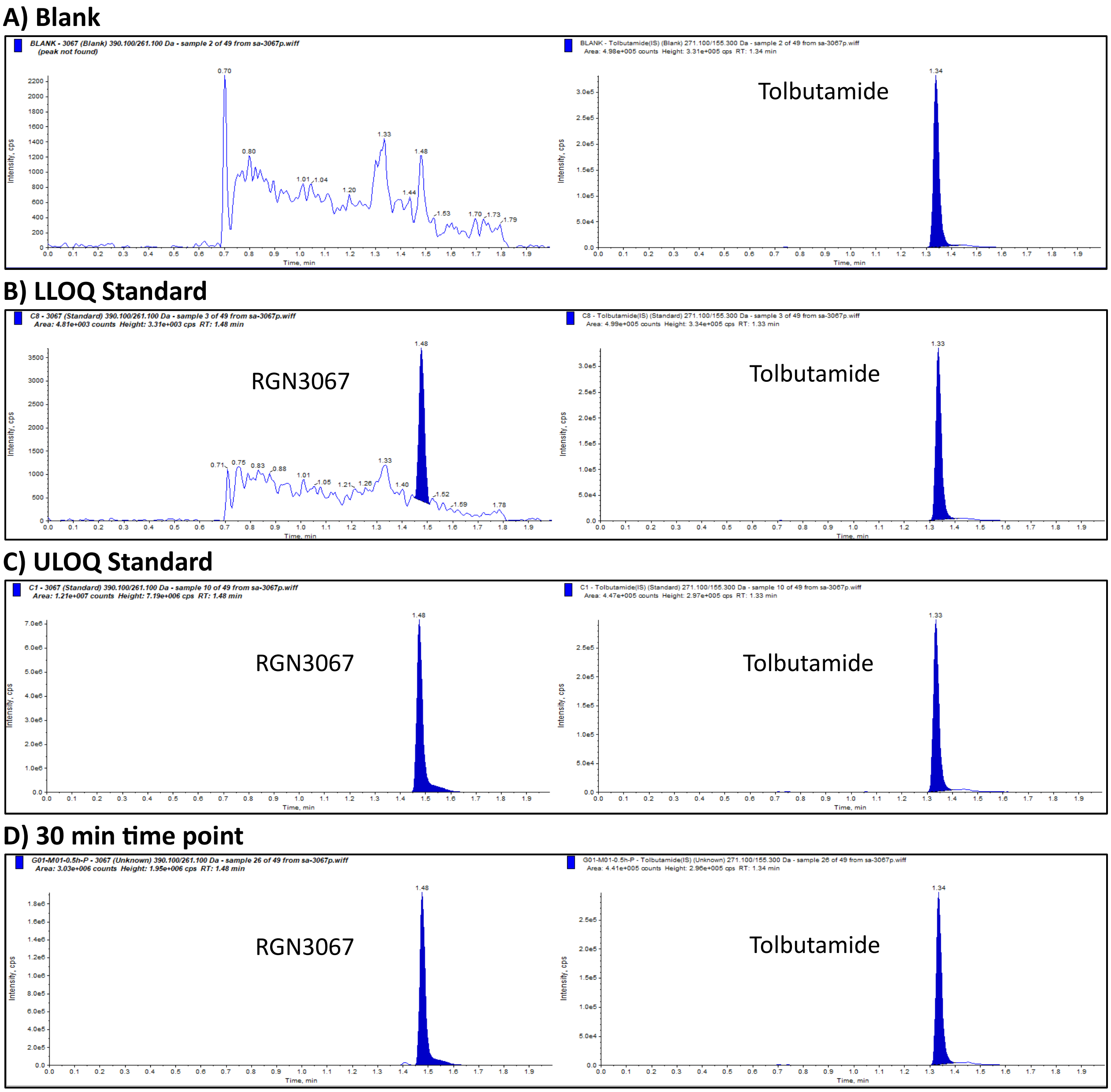

Figure S4. Representative LC-MS/MS chromatograms for the pharmacokinetic study on RGN3067 at the 30 min time point (mouse plasma). (A–C) Chromatograms of the blank, lower limit of quantification (LLOQ) and upper limit of quantification (ULOQ) samples. (D) The 30 min time point after administration of an oral dose of 100 mg/kg of RGN3067 (left panel). Tolbutamide was used as the internal standard (right panels, A–D). The data is auto-scaled.

A) Blank

B) LLOQ

C) ULOQ

D) 1 h time point

E) 8 h time point

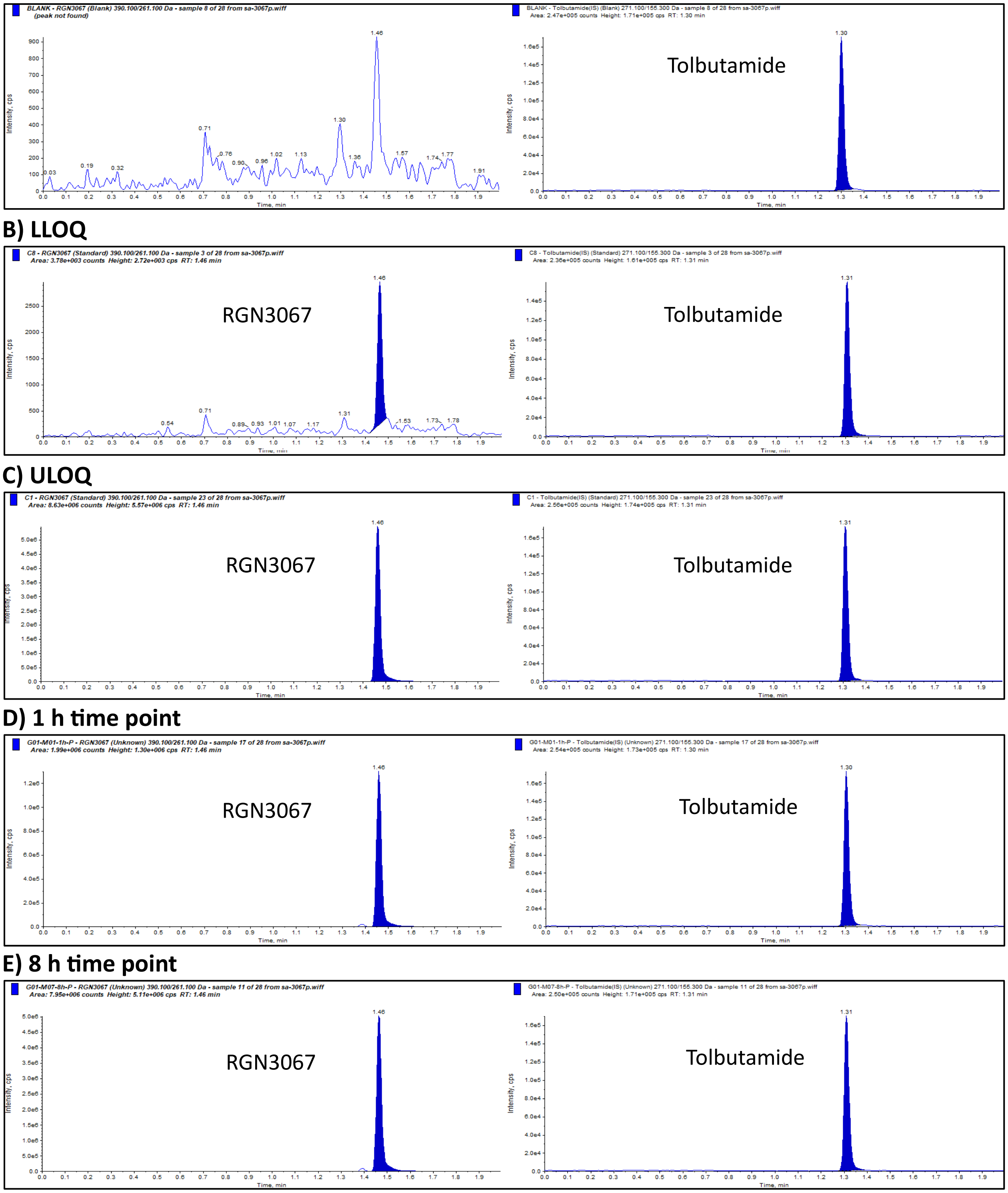

Figure S5. Representative LC-MS/MS chromatograms for the brain permeability study on RGN3067 at 1 and 8 h time points (mouse plasma). (A–C) Chromatograms of the blank, lower limit of quantification (LLOQ) and upper limit of quantification (ULOQ) samples. (D–E) The 1 and 8 h time points after administration of an oral dose of 100 mg/kg of RGN3067 (left

panels). Tolbutamide was used as the internal standard (right panels, A–E). The data is auto-scaled.

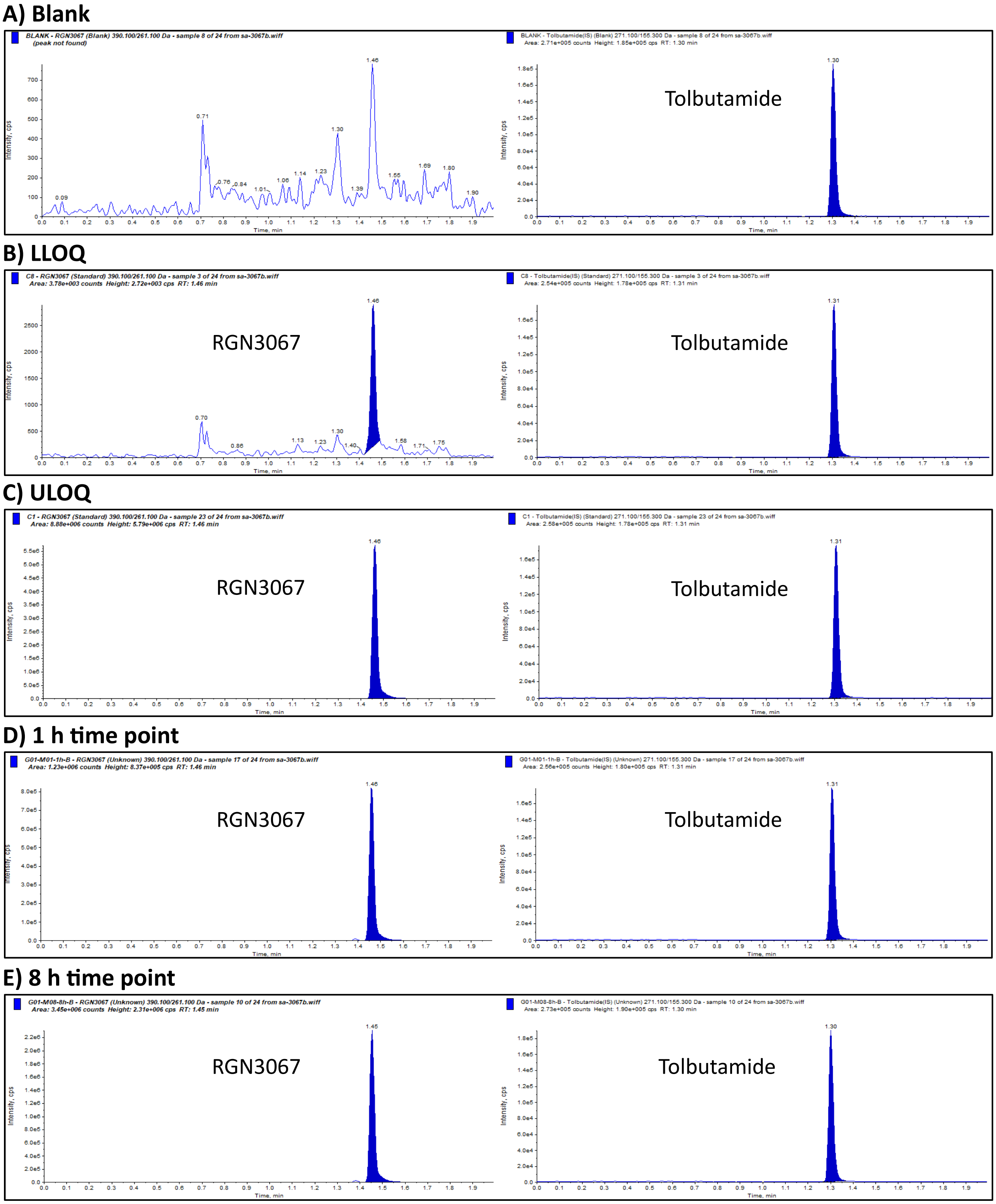

Figure S6. Representative LC-MS/MS chromatograms for the brain permeability study on RGN3067 at 1 and 8 h time points (mouse brain). (A–C) Chromatograms of the blank, lower limit of quantification (LLOQ) and upper limit of quantification (ULOQ) samples. (D–E) The 1 and

8 h time points after administration of an oral dose of 100 mg/kg of RGN3067 (left panels). Tolbutamide was used as the internal standard (right panels, A–E). The data is auto-scaled.

Kinetic solubility (μM)	PBS (pH = 7.4)	5.7
Cellular permeability (MDR1-MDCK)	Papp (A-B) 10^{-6} (cm/s)<br>Efflux ratio	22.7<br>0.61
Plasma protein binding (%)	Mouse<br>Rat<br>Human	97.4<br>97.4<br>98.2
Liver microsomal stability ($T_{1/2}$ – min)	Mouse<br>Rat<br>Human	34.0<br>17.4<br>86.9

Table S1. **eADME profile of RGN3067**. In the MDR1-MDCK cell permeability model, an efflux ratio <2 is consistent with a lack of efflux pump susceptibility. A Papp value >20×10^{-6} cm/s is consistent with good cellular permeability.

Metabolite Code	$[M+H]^+$ / $[M+NH_4]^+$ *m/z*	RT (min)	Relative Abundance (UV Peak Area %Total)				
			Mouse	Rat	Dog	Monkey	Human
M1	294.0637	11.86	2.08	1.33	14.44	1.46	+
M2	427.0900	12.82	9.29	+	+	+	+
M3	469.1004	12.90	+	6.88	ND	+	+
M4	235.0630	13.20	0.32	+	2.26	1.14	0.33
M5	*407.1114	14.04	3.35	4.72	1.74	0.61	0.29
M6	278.0689	15.21	0.92	2.45	15.62	14.57	6.14
M7	408.0951	15.93	9.36	35.62	14.22	58.72	73.73
M8	251.0579	16.31	4.88	+	+	+	+
M9	376.1054	17.42	+	+	10.73	0.92	0.73
M10	376.1056	17.71	+	+	ND	2.20	+
RGN3067	390.1210	21.30	69.82	49.01	40.98	20.39	18.78

Table S2. Metabolite identification of RGN3067. Abundance of RGN3067 and metabolites after incubation with mouse, rat, dog, monkey, and human hepatocytes. These are semi-quantitative data determined based on the UV response peak area at 236–276 nm; NA: Not applicable; ND: Not detected; +: Only detected by MS; *: Value of $[M+NH4]^+$.

IC_{50} Values (nM)				
**Cell Line**	**RGN3067**	**RGN3062**	**RGN3096**	**Colchicine**
U87	560 ± 238	588 ± 106	>10000	10 ± 5
LN-18	117 ± 110	73 ± 10	2431 ± 171	11 ± 7
GBM12*	148	NT	NT	NT
GBM15*	347	NT	NT	NT
GBM39*	326	NT	NT	NT
GBM43*	617	NT	NT	NT

Table S3. RGN compounds are active in glioblastoma cell lines. Compounds were incubated with U87 and LN-18 cells for 72 h or with patient-derived GB cells* for 144 h prior to the cell viability assay. Absolute IC_{50} values were calculated and are denoted as mean ± SD from at least three independent experiments. *Data from patient-derived cells were collected at the Translational Genomics Research Institute (TGen). NT, not tested.

IC_{50} Values (nM)		
**Compounds**	**No Wash**	**Wash**
RGN3067	571 ± 171	>10000
Colchicine	13 ± 5	80 ± 45
Sabizabulin	11 ± 1	354 ± 69

Table S4. Reversibility experiments with RGN3067. IC_{50} values of compounds in the U87 glioblastoma cell viability assay after "washout" and "no-washout" conditions. Data are presented as mean ± SD and calculated from at least three independent experiments.

C_{max} (ng/mL)	7807 ± 1278
T_{max} (h)	2.00
$T_{1/2}$ (h)	3.61 ± 1.94
AUC_{0-last}(ng·h/mL)	51170 ± 7927
AUC_{0-inf} (ng·h/mL)	68614 ± 22139

Table S5. **Pharmacokinetics of oral administration of RGN3067 in CD-1 mice.** $C_{max,}$ maximum concentration in plasma; $T_{max,}$ time to reach maximum concentration; $T_{1/2}$, terminal half-life; AUC_{0-last}, AUC from time zero to the time of the last quantifiable concentration; AUC_{0-inf}, AUC from time zero extrapolated to infinity. Parameters are presented as mean ± SD.

Time (h)	Plasma (ng/mL)	Brain (ng/g)
1	3273 ± 2048	3570 ± 2356
4	2897 ± 533	2714 ± 972
8	7930 ± 5381	4842 ± 2722

Table S6. **Plasma and brain levels of RGN3067 after a 100 mg/kg oral dose in CD-1 mice**. Measurements were taken at 1, 4, and 8 h. Data are presented as mean ± SD.
